# Impact of Vitamin D Supplementation on Bone Mineral Density and All-Cause Mortality in Heart Transplant Patients

**DOI:** 10.3390/biomedicines9101450

**Published:** 2021-10-12

**Authors:** Mahmoud M. A. Abulmeaty, Deema A. Almutawa, Nedim Selimovic, May Almuammar, Abdulaziz A. Al-Khureif, Mohamed I. Hashem, Heba M. Hassan, Doaa A. Abdel Moety

**Affiliations:** 1 Department of Community Health Sciences, College of Applied Medical Sciences, King Saud University, Riyadh 11362, Saudi Arabia; deema.almutawa@gmail.com (D.A.A.); malmuammar@ksu.edu.sa (M.A.); dr.hebazaher@gmail.com (H.M.H.); 2 Medical Physiology Department, Faculty of Medicine, Zagazig University, Zagazig 44519, Egypt; daabdelmoeti@zu.edu.eg; 3 Health Sciences Department, Princess Nourah Bint Abdulrahman University, Riyadh 11564, Saudi Arabia; 4 King Faisal Specialist Hospital and Research Center, Riyadh 12713, Saudi Arabia; selimovicne@ngha.med.sa; 5 King Abdulaziz Cardiac Centre, Department of Cardiology, Riyadh 12713, Saudi Arabia; 6 Department of Molecular and Clinical Medicine, University of Gothenburg, 40530 Göteborg, Sweden; 7 Dental Health Department, Dental Biomaterials Research Chair, College of Applied Medical Sciences, King Saud University, Riyadh 10219, Saudi Arabia; aalkhuraif@ksu.edu.sa (A.A.A.-K.); mihashem@ksu.edu.sa (M.I.H.)

**Keywords:** vitamin D, bone mineral density, supplementation, all-cause mortality, heart transplant

## Abstract

Vitamin D (VD) deficiency is frequently reported in heart transplant (HT) recipients and routinely supplemented. However, the efficacy of VD supplementation on bone mineral density (BMD) and its association with all-cause mortality is underinvestigated. The VD levels and BMD were studied for two years, and the association of VD and BMD with all-cause mortality risk was investigated. Ninety-six HT patients (38.18 ± 12.10 years old; 74% men) were followed up during VD, Ca, and Mg supplementation. Anthropometric measurements, BMD by Dual-energy X-ray absorptiometry (DEXA) scan, VD concentrations, and related biochemical parameters were analyzed before, 1 year, and 2 years after HT. Despite significant improvement of VD_3_ and 25-hydroxy VD (25OHVD) levels especially in the men, BMD parameters were insignificantly changed. After 2 years, the all-cause mortality rate was 15.6%. High pretransplant levels of 25OHVD failed to improve the survival probability. Cox’s regression showed a 32.7% increased hazard ratio for each unit increase in body mass index (95% CI: 1.015–1.733, *p* = 0.038), in the VD-deficient group rather than in the VD-sufficient one. In conclusion, VD supplementation improves the biochemical status, especially in VD-deficient HT. However, its impact on the BMD and mortality was not as usually expected. Further investigation of the disturbed VD metabolism in HT is warranted.

## 1. Introduction

Vitamin D deficiency (VD-D) is highly prevalent among patients with end-stage organ failure especially heart failure. Further, VD-D is frequently reported in heart transplant (HT) patients [1,2]. Patients eligible for HT are hardly exposed to sunlight due to frequent hospitalization and defective hepatic metabolism for VD due to heart failure-associated hepatic congestion. The VD status is frequently represented by the level of 25 hydroxyvitamin D (25OHVD). The International Osteoporosis Foundation (IOF) reported the definition of the VD-D by having a serum level of 25OHVD less than 25 nmol/L [3]. However, many cutoff points for defining VD-D were also reported, e.g., serum level of 25OHVD < 30 nmol/L [4], and 25OHVD < 50 nmol/L [5]. The 25 nmo/L was suggested as an arbitrary cutoff value especially with a lack of sufficient evidence for the optimal value for non-skeletal effects of VD [6,7], especially in populations with endemic VD-D such as Saudi Arabia. In Saudi Arabia, VD-D (with 25 nmol/L cutoff point) was reported as 44.5% in adults and 49.5% in school children [8]. Heart transplant is still growing in Saudi Arabia, with only 2 cardiac centers available to do HT with a rate of about 30 HT/year [9]. In a recent study, VD-D (reported by 25OHVD < 25 nmol/L) was reported in 10% of the heart transplant patients and 55% of those with orthotopic heart transplants [10]. However, the data about VD status, bone mineral density (BMD), and the impact of VD on survival are deficient.

Osteopenia or osteoporosis may develop in HT patients especially in the first year and with improper preventive management [11]. Glucocorticoids bind osteoprotegerin (OPG); this molecule is necessary for limiting bone resorption. Thus, the decreased amount of OPG reduces BMD in transplantation patients [12]. Notably, it was reported that patients referred to cardiac transplantation generally have low BMD and about 14% of them suffer from osteoporotic vertebral compression fractures [13]. In another report, the prevalence of vertebral fractures was reported as 35% after HT heart transplantation due to increased bone loss [12]. Previous reports from our center showed osteopenia (35%) and osteoporosis (8%) at the lumbar spine of pre-transplant patients, besides significant reduction in pre-transplant BMD compared with that at 1 year after heart transplantation [14]. Nutritional supplementation with VD, Ca, Mg, Zn, and vitamin C are frequently recommended to support BMD in HT patients [2,15]. The International Society for Heart and Lung Transplantation recommended a daily calcium dose of about 1000–1500 mg, and VD 400–1000 IU for HT patients to maintain serum 25OHVD level > 75 nmol/L but with a low level of evidence [16].

The impact of VD and low BMD on all-cause mortality in HT patients is underinvestigated. Some reports showed that a reduced level of 1,25(OH)_2_VD, measured on the 21st day after HT, was associated with 1-year mortality in HT recipients [17]. This relationship between 1,25(OH)_2_VD (calcitriol) and mortality, is unclear. Low 1,25(OH)_2_VD may be because of immunosuppressive medications such as calcineurin inhibitors on the renal enzyme system, or due to bad general health as a consequence following organ transplantation. In another study that used the most common indicator of VD status monitoring (25OHVD), VD supplementation in a dose of 4000 IU did not affect the mortality in patients with end-stage heart failure compared to placebo [18]. Another European randomized controlled trial (NTC01212406) used VD in a high dose of 100,000 IU/month in lung transplantation patients and reported low survival for patients who received VD [19]. Data from another area on the map such as Saudi Arabia are very lacking. Thus, this work aimed to study the changes in VD levels and BMD in VD-supplemented HT patients for two years and to analyze the association of VD levels and BMD with the survival of Saudi HT recipients.

## 2. Materials and Methods

### 2.1. Study Design and Setting

A total of ninety-six heart transplant patients at King Faisal Specialist Hospital & Research Centre (KFSH&RC) in Riyadh, Saudi Arabia were investigated and followed up for two years after the procedure had been done. Participants were divided into two groups based on the baseline level of 25OHVD: Group I in which 25OHVD < 25 nmol/L, and Group II with 25OHVD ≥ 25 nmol/L. The Research Ethics Committee (REC) in the College of Applied Medical Sciences, King Saud University reviewed and approved this study protocol under reference number CAMS 93-36/37; date: 01/03/2016. In addition, the REC at KFSH&RC approved it under reference No. 2161051.

### 2.2. Given Supplementations and Medications

**Figure 1:**
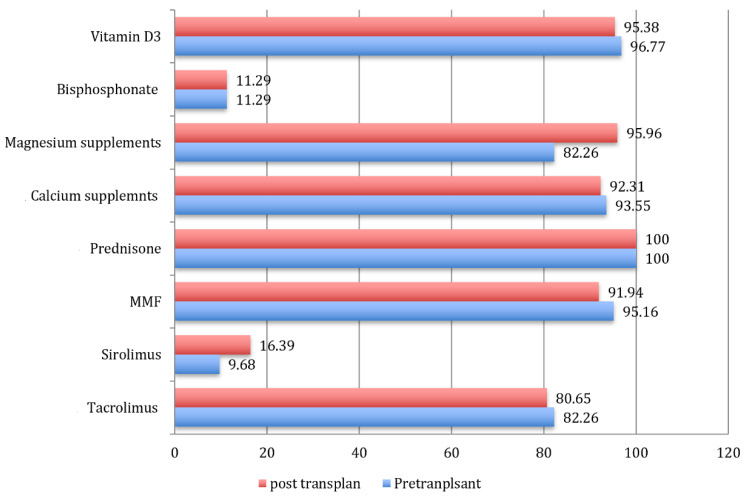
Medications intake by study population in the pre-transplant and post-transplant phases (numbers refer to frequencies of medication use, *MMF* = mycophenolic acid).

Study participants were on medications as seen in Figure 1. According to the KFSH&RC’s local protocol, a routine dose of vitamin D_3_ was 10,000 IU (250 μg/day) for patients with VD insufficiency (25OHVD < 50 nmol/L), while those with proven VD-D (by 25OHVD < 25 nmol/L) were given 50,000 IU as a weekly oral dose for 3 months followed by a 10,000 IU maintenance dose. Calcium (Ca) in an oral dose of 1200 mg/day, and magnesium (Mg) 200 mg/day per oral were also routinely given to all patients. Commitment percentages of study participants on other supplements and medications were shown in Figure 1.

### 2.3. Anthropometric Parameters

Bodyweight (kg) and height (cm) were used for calculating body mass index (BMI) using the formula; BMI = weight (kg)/height (m)^2^. Weight was measured to the nearest 0.1 kg by (Scale-Tronix scale, Chicago, IL, USA) while a stadiometer (Seca Co, Hamburg, Germany) was used for height measurement.

### 2.4. Measurement of VD and Biochemical Parameters

Serum cholecalciferol (VD_3_), 25OH vitamin D (25OHVD), and intact parathyroid hormone (iPTH) levels (pmol/L) were measured on pretransplant assessment workup appointment, one year, and two years after the transplant had done by using electrochemiluminescence immunoassay, Cobas e411 autoanalyzer (Roche Ltd., Basel, Switzerland). Despite previous reports, the definition of VD-D was suggested in this study to be below 25 nmol/L of the 25OHVD level [20,21,22]. Moreover, levels of Alkaline phosphatase (ALP) (U/L), calcium (mmol/L), phosphorus (mmol/L), magnesium (mmol/L), creatinine (umol/L), urea (mmol/L), potassium (mmol/L), and chloride (mmol/L) were measured by Roche/Hitachi modular Cobas c 701/702 tests.

### 2.5. Measurement of BMD

The BMD was measured at scheduled appointments for follow-up at pre-transplant, 1 year, and 2 years post-transplant phases and the reports were used for analysis. BMD was assessed by the DEXA scan using a GE medical system Lunar iDEXA (GE Healthcare, Madison, WI, USA). Two sites were selected: lumbar spine (LS) and femoral neck (FN). Due to the relatively young age of our samples, Z-scores (rather than T-scores) were calculated as standard deviations from the mean of the gender- and age-matched controls. Tertials of the BMD results were created as follows: (a) normal BMD: Z-scores above −1; (b) osteopenia: Z-scores between −1 and −2.5 gm/cm^2^; and (c) osteoporosis: Z-score below −2.5 gm/cm^2^ [23].

### 2.6. Sample Size and Satistical Power

The sample size and statistical power were calculated by G*Power software 3.1.9.4 (University of Kiel, Kiel, Germany), considering medium effect size (f = 0.25), alpha error probability at 0.05, power (1-β error probability) = 0.95, number of repeated measurements = 3, and the number of groups = 2. The estimated total sample size was 44 participants (22/group) and the actual power was 0.9557.

### 2.7. Statistical Analysis

The SPSS tool version 25 (SPSS, IBM, Chicago, IL, USA) was used for processing and analyzing these data. Continuous data were expressed as means ± SD, while dichotomous variables were expressed as percentages and categories. The normal distribution of continuous variables was tested by the Shapiro–Wilk test. For comparison of the three related samples, the Friedman ANOVA test was used with pairwise comparisons. Gender differences and comparisons between groups I and II were done via the Mann–Whitney U test. For survival analysis, Cox’s proportional hazard regression analysis was used with 95% CIs. Results were considered statistically significant at *p* ≤ 0.05.

## 3. Results

### 3.1. Basal Characteristics of Study Participants

**Figure 2:**
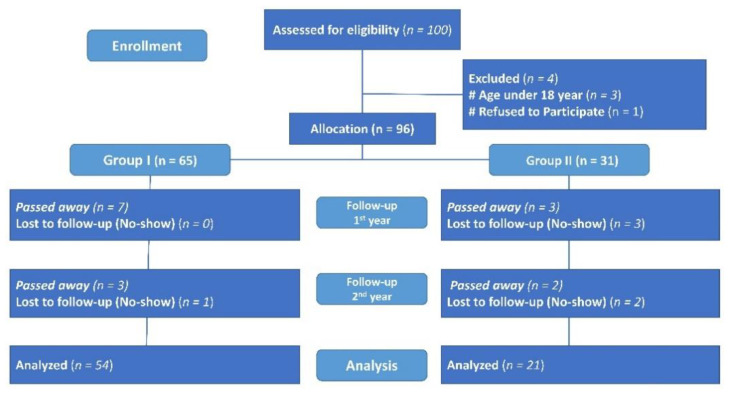
CONSORT diagram showing participants’ flow throughout the study.

**Figure 3:**
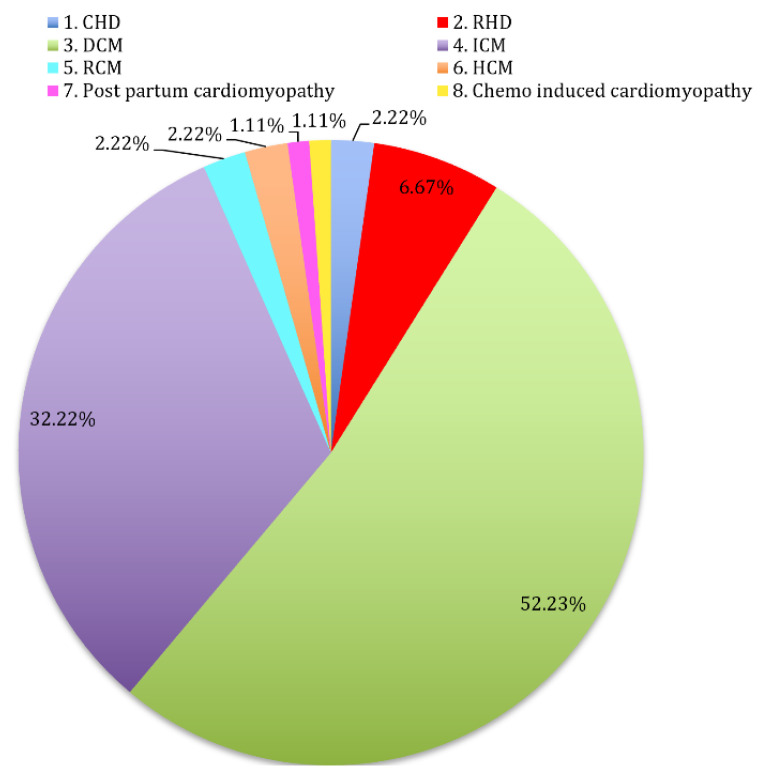
Percentages of main pre-transplant diagnoses among study recipients. (CHD: congenital heart disease, RHD: rheumatoid heart disease, DCM: dilated cardiomyopathy, ICM: ischemic cardiomyopathy, RCM: restrictive cardiomyopathy, HCM: hypertrophic cardiomyopathy).

Figure 2 shows participants’ flow throughout the study. As shown in Table 1, pretransplant data of our sample showed younger age, significantly lower BMI, and BMD in the women’s group. Levels of VD_3_, 25OHVD, PTH, Ca, ALP, Urea, Na, and K were insignificantly different. However, Mg and Cl levels were significantly higher, while creatinine was lower in the women group. Figure 3 shows the cardiovascular events which were diagnosed in our participants. The majority had dilated cardiomyopathy (52.22%), followed by ischemic cardiomyopathy (32.22%), and chemo-induced cardiomyopathy and post-partum cardiomyopathy (1.11%).

### 3.2. Changes in VD and BMD throughout the Study Period

**Figure 4:**
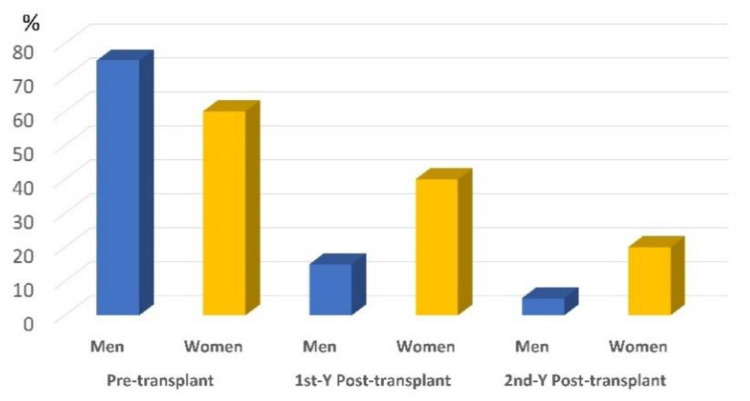
Prevalence of VD deficiency defined by 25OHVD < 25 nmol/L in men and women’s groups throughout the study period.

**Figure 5:**
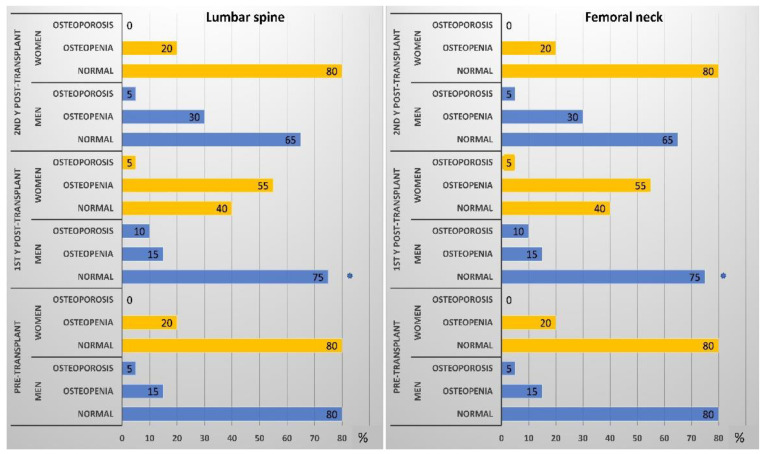
Prevalence of osteopenia and osteoporosis in men and women groups throughout the study period (* means significantly different between men and women by Mann–Whitney U test).

**Figure 6:**
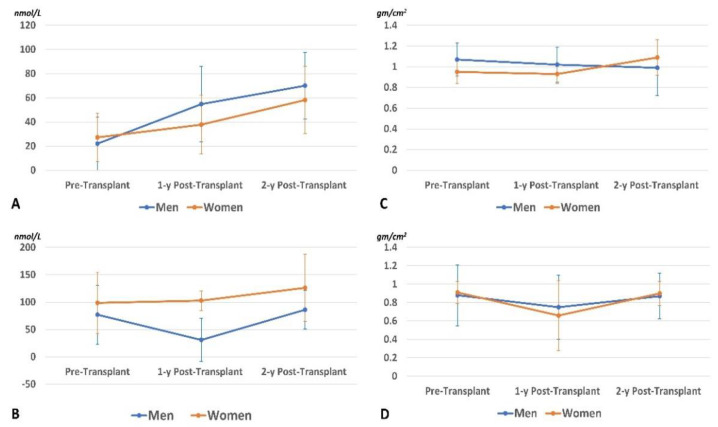
Longitudinal changes in the 25OHVD level (**A**), PTH level (**B**), BMD at the lumbar spine (**C**), and femoral neck (**D**).

After 2 years, 15 participants passed away (33% women), and 6 dropped out (83% women). The data of remaining participants (*n* = 75; 20% women) are presented in Table 2 and analyzed by the Friedman ANOVA test with pairwise comparisons. Bodyweight and BMI significantly improved, indicating improvement of nutritional status. Femoral BMD in the men’s group showed a significant reduction after 1 year. After the second year, it returned to a value like that of the pretransplant status. In women, the three measurements were insignificantly different (*p* trend > 0.05). In the men’s group, levels of 25OHVD and Ca increased progressively throughout the period with a significant reduction of PTH (especially in the first year), and ALP enzyme. However, insignificant changes in their levels were noticed in the women group. The prevalence of VD-D (defined by 25OHVD < 25 nmol/L) is presented in Figure 4. A progressive reduction of the VD_D was noticed, especially in the men’s group, indicating sufficient VD supplementation. Despite supplementation, Mg serum level showed progressive reduction (*p* < 0.05) in men and women (*p* = 0.74). Percentages of study participants with osteopenia and osteoporosis at the lumbar spine and femoral neck throughout the study period are presented in Figure 5. Longitudinal changes in the 25OHVD, and PTH, as well as BMD at the lumbar spine and femoral neck, are shown in Figure 6.

Table 3 further reports the changes in study parameters in both study groups. Pretransplant body BMI was insignificantly different between group I and group II. However, at the post-transplant assessment points, the BMI was significantly higher in the VD-sufficient group (group II). Besides, longitudinal changes showed a progressive increase of BMI with time, especially in the VD-deficient group. Group I had significantly lower VD_3_, 25OHVD, and calcium levels. In Group I (VD-D group), significant reductions in BMD parameters were detected after the first year which improved at the second year to be insignificantly different from the pretransplant levels. In Group II, insignificant changes were reported in the three-time points. VD_3_ level progressively increased with time due to the supplementation, while the 25OHVD level significantly increased in group I rather than group II. Similarly, levels of iPTH, ALP, calcium, and magnesium showed significant changes in group I rather than group II.

### 3.3. Survival Analysis Based on VD and BMD

Notably, in Group I with VD-D, Cox’s regression analysis showed that for each additional unit of BMI, the hazard increases by about 33% (Table 4). This was not the case in Group II. Besides, in both groups, for each additional unit of 25OHVD or VD_3_, the hazard ratio (HR) showed insignificant changes (Table 4). Furthermore, the age and BMD parameters had insignificant impacts.

## 4. Discussion

This study investigated changes in the VD levels and BMD in HT patients for two years and analyzed the association of VD status (by using an arbitrary cutoff value of 25OHVD, i.e., 25 nmol/L), and BMD with all causes-mortality in vitamin D-deficient and -sufficient groups of HT Saudi recipients. Most of our study participants were on VD supplementation at least by a maintenance dose of 10,000 IU/day. This was successful in the reduction of the percentage of the VD deficiency in both men and women’s groups (Figure 2). Besides, means of the 25OHVD and VD_3_ serum levels were significantly increased progressively in the men’s group and Group I, while the rise of their levels in the women’s group and group II were insignificant. Indicating that the benefit of VD supplementation is prominent in those with VD-D. Our baseline percentage of the VD-D was much higher than that reported by Stein et al. [24] where severe deficiency (25OHD <25 nmol/L) was found in 16% of heart transplant patients. Moreover, a Slovenian cohort of HT recipients showed 21.3% with severe VD deficiency and 54.7% with mild-to-moderate VD-D. However, these patients were on VD_3_ supplementation in a dose of 2000 IU/day and Alfacalcidol of 0.5 μg/day [25]. This frequently reported phenomenon is critical, since the VD-D is linked to post-HT bone loss and fracture possibility, sarcopenia, and may aggravate the immunosuppressive action of corticosteroids or calcineurin inhibitors [26]. Besides, it is associated with periodontal disease and gingival inflammation in HT recipients which impair the nutritional intake [27]. The improvement of VD status after HT especially in the VD- and Ca-supplemented men was in line with a previous report by Gilfraguas et al. [28]. Supplementation in addition to relief of hepatic congestion and improvement of general condition with more mobility and sunlight exposure were the causes of VD status improvement [29]. Unfortunately, this was not the case in the women’s group especially in Saudi Arabia where indoor lifestyle and extensive body covering are the traditions.

Bone metabolism and VD status are closely related. Low 25OHVD levels can negatively impact bone turnover biochemical markers. The PTH as an indicator of bone resorption is usually investigated. Baseline measurements of PTH in our sample indicated higher serum levels of iPTH and low normal Ca levels together with low 25OHVD (Table 2 and Table 3). This secondary hyperparathyroidism was improved in the men’s group and the deficient groups after the first year then relapsed later, while in Group II and women, insignificant changes were detected in the first year and a significant increase in iPTH (with insignificant reduction of Ca level) were detected by the second year. This finding was consistent with previous reports [27,29]. The increase in the 25OHVD level leads to normalization of serum calcium and phosphate levels, nevertheless, serum iPTH level remained high, especially in the women’s group and in the second year in the men’s group, indicating a status of persistent hyperparathyroidism in the HT recipients. This persistent secondary hyperparathyroidism occurred in both deficient and sufficient groups in the second year. This finding was consistent with previous reports about HT [30,31], renal transplant adults [32], and up to 50% of children’s kidney transplants [33]. Persistent secondary hyperparathyroidism may then lead to autonomous hyperplasia of parathyroid glands. Besides, perioperative administration of large amounts of citrate during blood transfusion leading to precipitation of calcium resulting in hypocalcemia-induced hyperparathyroidism. This persistent hyperparathyroidism or tertiary hyperparathyroidism is usually reported after successful renal transplantation. PTH levels usually decline significantly within the first 3–6 months after kidney transplantation due to the reduction of the functional mass of parathyroid glands [34]. Persistent hyperparathyroidism despite normalization of renal functions, and overall survival was reported in 25% of kidney transplant recipients 1-year after the procedure. Medical management and even parathyroidectomy may be required in these cases [35,36,37,38,39,40]. Moreover, it may cause serious consequences such as hypercalcemia, organ calcification, hypophosphatemia, and hypercalciuria [34,41].

Despite VD and Ca supplementation, a significant reduction in femoral neck BMD after 1 year was noticed especially in group I, while all remaining measurements were insignificantly different from the pretransplant status, especially in Group II and women. These findings were consistent with previous reports [13,42] about both lung and heart recipients. Compared to pretransplant status, Caffarelli et al. [42] found an increase in the incidence rate of vertebral fractures in the first period post-transplantation (9.6% vs. 25.7%). These vertebral fractures were predicted only by the history of any fracture, while in lung transplant recipients, vertebral fractures were predicted by age, BMD at the femur neck, and history of fracture [43]. The transplantation-associated abnormalities in bone metabolism are generally similar regardless of the transplanted organ, pre-existent low BMD, and previous treatment. Typically, bone loss occurs in the first year after the organ transplant, because of immunosuppressive medications, and the long period of immobilization. Supplementation with VD, Ca, and Mg was not sufficient in the improvement of BMD in the HT population. At least in part, tertiary hyperparathyroidism may be the underlying mechanism. In another hand, Calcitriol (1,25(OH)2VD) supplementation in a dose of 0.5–0.75 µg/day for 12 or 24 months in addition to calcium 600 mg/day in comparison with calcium 600 mg/day produced improvement in the femoral neck (but not at lumbar spine) in the calcitriol groups at 12 months [44]. In another trial, Calcidiol (25OHVD) in a dose of 32,000 IU/week showed a mild improvement of about 4.9% only at the lumbar spine in HT patients [45]

In our study ALP, and urea significantly decreased in the male group rather than the women’s group (Table 2). In the pretransplant phase, congestive hepatopathy and even liver cirrhosis may be evident resulting in impaired hepatic functions such as protein and lipid biosynthesis and decreased ability for detoxification of toxic metabolites. Besides, secretions of hepatic enzymes show an abnormal pattern such as rises in alkaline phosphatase (ALP), gamma-glutamyl transferase (GGT), aspartate aminotransferase (AST), and alanine aminotransferase (ALT) [46]. Post-transplant relief of hepatic congestion greatly correct this abnormal pattern. Moreover, Przybyłowski et al. [10] reported that vitamin D was correlated with kidney functions in heart transplant patients, i.e., improvement of VD status was associated with improvement of renal functions.

Interestingly, Cox’s regression analysis showed that in Group I, for each additional unite of 25OHVD, the hazard for all-cause mortality decreases by 7% (HR = 0.930; 95%CI: 0.681–1.270, *p* = 0.648), while in Group II, for each additional unite of 25OHVD, the hazard decreases by 65.5% (HR = 0.345; 95%CI: 0.650–1.163, *p* = 0.345). However, these findings were statistically insignificant (Table 4). The current study finding was in line with Zittermann et al. [18] who reported no effect of 4000 IU/day oral vitamin D supplementation in reduction of mortality in patients with advanced heart failure. After 3 years there was no beneficial latency impact of VD supplementation on all-cause mortality in the same study participants [47]. In a meta-analysis of randomized clinical trials, with >83,000 participants, VD supplementation failed in reducing the risks of major adverse cardiovascular events, stroke, myocardial infarction, cardiovascular disease mortality, or all-cause mortality [48]. In children undergoing hematopoietic stem cell transplant, there was no significant difference in overall survival for those with pretransplant VD deficiency, or sufficiency or optimal level (*p* = 0.51) [49]. In a renal transplant study, Cox regression analysis showed no significant prediction between 3-month 25OHVD or 3-month 1,25(OH)_2_VD levels and mortality (HR = 0.97; 95% CI: 0.93–1.02, *p* = 0.27 for 1 25OHVD unit increase, and for 10 units increase it was 0.86; 95% CI: 0.70–1.06, *p* = 0.16) [50]. On the other hand, Zittermann et al. [17] found that low postoperative levels of 1,25(OH)_2_VD were associated with high 1-year mortality in HT recipients. Furthermore, in a large cohort of kidney transplant recipients, survival was better in recipients with sufficient vitamin D which was measured 10 weeks post-transplant [51]. However, we used a different indicator of the VD status (i.e., 25OHVD), and we used the pretransplant level. Another report from patients with chronic heart failure showed about a 14% reduction of all-cause mortality with a 2.7-fold increment in the 25OHVD Level (95% CI: 1–26%; *p* = 0.04) [52]. Pediatric reports also stated that VD-D was associated with lower survival on a short-term basis after hematopoietic stem cell transplantation in children [53].

This study’s findings indicate a significant increase in the HR of all-cause mortality in the VD-deficient group rather than the VD-sufficient group (HR = 1.327; 95% CI: 1.015–1.733, *p* = 0.038). Independent of the VD status, a systematic review showed that pretransplant BMI was associated with increased risk of mortality in those with BMI above 30 Kg/m^2^ (10% increase in HR) and those above 35 kg/m^2^ (by about 24%) [54]. Moreover, Doumouras et al. [55] added the low BMI to the obesity as independent factors for increased mortality in HT recipients. While Nagendran et al. [56] excluded BMI up to 35 kg/m^2^ from the factors that worse mortality risk. Our sample’s pretransplant BMI was at the range of normal BMI in Group I, and the overweightedness in Group II. In the general population, obesity may affect the association between VD and cardiac disorders. However, VD supplementation failed to reduce the incidence rate of cardiovascular diseases and mortality [57]. In the HT population, the current study tested the BMI and VD levels in the same Cox’s regression model and resulted in a significant effect of BMI with an insignificant effect of the VD levels. This indicates that the mortality-increasing effect of the BMI is independent of VD.

## 5. Conclusions

This study tracked the changes of the VD3, 25OHVD, and BMD in VD-supplemented heart transplant recipients for 2 years. Further, it investigated the association of 25OHVD, and BMD with all-cause mortality, based on an arbitrary cutoff value of 25OHVD equal 25 nmo/L. Supplementation with VD_3_ 10,000 IU daily dose, Ca 1200 mg/day, and Mg 200 mg/day were being effective in elevating the serum level of 25OHVD especially in vitamin D deficient HT recipients; however, no significant impact was detected on the preservation of the BMD at measured sites, or on correction of tertiary hyperparathyroidism. Interestingly, the 25OHVD failed to ameliorate the all-cause mortality hazard ratio.

## 6. Limitations

The main limitation of this study was the lack of a placebo-controlled group. Instead, we used the comparison of VD deficient (Group I) vs. VD sufficient (Group II). The limited number in the women’s group is also a considerable limitation that may affect the obtained results. However, the number of female candidates is usually less than males in many centers all over the world. Another important limitation is the missing of about 21.8% of our participants at the end of the study; either by death (15.6%) or by no-show (6.25%). Missing data are usually common in longitudinal studies. Besides, we did not measure the 1,25(OH)2VD levels and considered the commonly used indicator for VD status which is 25OHVD.

## Figures and Tables

**Table 1:** Pretransplant characteristics of the study sample.

Variables	Total (*n* = 96)	Men (*n* = 71)	Women (*n* = 25)	*p*-Value
Age (Years)	36.17 ± 13.53	39.84 ± 12.22	32.35 ± 9.31	0.031
Height (cm)	165.33 ± 9.02	168.56 ± 7.57	156.16 ± 6.02	<0.001
Weight (kg)	65.05 ± 17.29	69.68 ± 15.87	51.90 ± 14.31	<0.001
BMI (kg/m^2^)	23.62 ± 5.37	24.50 ± 5.21	21.12 ± 5.11	0.006
BMD lumbar spine (gm/cm^2^)	1.05 ± 0.16	1.07 ± 0.16	0.95 ± 0.11	0.041
Lumbar spine Z-score	−0.31 ± 1.10	−0.25 ± 1.15	−0.48 ± 0.96	0.380
BMD femoral neck (gm/ cm^2^)	0.59 ± 0.49	0.67 ± 0.48	0.38 ± 0.45	0.012
Femoral neck Z-score	−0.10 ± 0.91	−0.04 ± 0.99	−0.26 ± 0.60	0.293
25OHVD (nmol/L)	27.80 ± 23.78	27.72 ± 24.63	28.04 ± 21.63	0.954
Vitamin D_3_ (nmol/L)	14.82 ± 14.73	15.23 ± 13.63	13.68 ± 17.76	0.654
Intact parathyroid h. (mmol/L)	78.15 ± 65.13	80.17 ± 61.88	72.40 ± 74.67	0.611
ALP (U/L)	96.07 ± 78.76	99.97 ± 80.56	84.98 ± 73	0.416
Calcium (mmol/L)	2.22 ± 0.18	2.20 ± 0.18	2.28 ± 0.19	0.054
Phosphate (mmol/L)	1.14 ± 0.34	1.15 ± 0.34	1.11 ± 0.35	0.600
Mg (mmol/L)	0.95 ± 0.55	0.86 ± 0.17	1.19 ± 1.02	0.011
Creatinine (umol/L)	91.14 ± 36.80	96.01 ± 36.14	77.32 ± 35.83	0.028
Urea (mmol/L)	10.14 ± 6.59	10.71 ± 5.75	8.50 ± 8.45	0.149
Sodium (mmol/L)	136.67 ± 6.43	136.04 ± 5.87	138.44 ± 7.63	0.110
Potassium (mmol/L)	4.05 ± 0.57	4.03 ± 0.56	4.10 ± 0.60	0.571
Chloride (mmol/L)	97.62 ± 6.89	96.52 ± 6.24	100.64 ± 7.80	0.010

BMI is body mass index; BMD is bone mineral density; 25OHVD is 25 hydroxyvitamin D; ALP is alkaline phosphatase; Mg is magnesium.

**Table 2:** Bone mineral density and related biochemical parameters before, 1 year, and 2 years after the heart transplant.

Variables	Male (*n* = 60)			*p*-Value ^†^	Female (*n* = 15)			*p*-Value ^†^
	Pre-Transplant Mean ± SD	1-Y Post-Transplant Mean ± SD	2-Y Post-Transplant Mean ± SD		Pre-Transplant Mean ± SD	1-Y Post-Transplant Mean ± SD	2-Y Post-Transplant Mean ± SD	
Weight (kg)	70.08 ± 13.24 ^a,^*	76.95 ± 14.95 ^b,^*	89.03 ± 12.40 ^b,^*	<0.001	52.41 ± 12.56 ^a^	61.89 ± 17.61 ^b^	64.35 ± 18.97 ^b^	0.015
BMI (kg/m^2^)	24.75 ± 4.43 ^a^	26.85 ± 5.37 ^b,^*	27.21 ± 5.15 ^b^	0.011	21.46 ± 5.20 ^a^	24.86 ± 6.52 ^b^	26.27 ± 7.27 ^b^	0.021
DEXA parameters								
BMD lumbar spine (gm/cm^2^)	1.07 ± 0.16 ^a,^*	1.02 ± 0.17 ^a,^*	0.99 ± 0.27 ^a,^*	0.231	0.95 ± 0.11 ^a^	0.93 ± 0.09 ^a^	1.09 ± 0.17 ^a^	0.247
Lumbar spine Z-score	−0.28 ± 1.49 ^a^	−0.66 ± 1.39 ^b,^*	−0.50 ± 1.47 ^a^	0.019	−0.12 ± 0.99 ^a^	−1.02 ± 0.59 ^b^	−0.80 ± 1.10 ^a^	0.016
BMD femoral neck (gm/cm^2^)	0.88 ± 0.33 ^a^	0.75 ± 0.35 ^b,^*	0.87 ± 0.25 ^a^	0.012	0.91 ± 0.12 ^a^	0.66 ± 0.38 ^a^	0.90 ± 0.13 ^a^	0.268
Femoral neck Z-score	−0.09 ± 1.33 ^a,^*	−0.56 ± 1.22 ^b,^*	−0.47 ± 1.18 ^a,^*	0.106	−0.10 ± 0.61 ^a^	−0.78 ± 0.75 ^b^	−0.36 ± 0.62 ^a^	0.007
Biochemical parameters								
25OHVD (nmol/L)	22.07 ± 22.02 ^a^	54.79 ± 31.13 ^b,^*	70.05 ± 27.58 ^b,^*	<0.001	27.20 ± 20.13 ^a^	37.80 ± 24.36 ^a^	58.20 ± 27.69 ^a^	0.143
Vitamin D_3_ (nmol/L)	17.07 ± 14.36 ^a^	40.44 ± 25.43 ^b,^*	64.00 ± 25.06 ^c^	<0.001	17.80 ± 18.83 ^a^	32.47 ± 19.65 ^a^	51.21 ± 25.19 ^a^	0.145
Intact parathyroid h (pmol/L)	77.40 ± 53.88 ^a,^*	30.85 ± 39.41 ^b,^*	86.09 ± 35.36 ^c^	0.013	98.67 ± 55.93 ^a^	102.80 ± 18.10 ^a^	126.25 ± 61.1 ^b^	0.015
ALP (U/L)	103.70 ± 42.42 ^a,^*	94.55 ± 42.85 ^a,^*	81.71 ± 29.10 ^b^	0.026	89.00 ± 40.14 ^a^	65.00 ± 12.63 ^a^	63.80 ± 4.55 ^a^	0.449
Calcium (mmol/L)	2.18 ± 0.14 ^a,^*	2.26 ± 0.11 ^b,^*	2.27 ± 0.9 ^b,^*	0.024	2.25 ± 0.12 ^a^	2.17 ± 0.04 ^a^	2.02 ± 0.50 ^a^	0.449
Phosphate (mmol/L)	1.10 ± 0.30 ^a,^*	1.12 ± 0.18 ^a,^*	1.07 ± 0.16 ^a^	0.314	1.00 ± 0.44 ^a^	1.09 ± 0.25 ^a^	1.01 ± 0.34 ^a^	0.531
Mg (mmol/L)	0.88 ± 0.12 ^a,^*	0.72 ± 0.10 ^b^	0.64 ± 0.18 ^b^	<0.001	0.91 ± 0.11 ^a^	0.71 ± 0.16 ^a^	0.62 ± 0.05 ^a^	0.074
Creatinine (umol/L)	95.85 ± 35.06 ^a,^*	108.25 ± 52.63 ^a,^*	95.67 ± 23.88 ^b^	0.819	79.00 ± 23.36 ^a^	80.40 ± 31.30 ^a^	79.00 ± 24.22 ^a^	0.437
Urea (mmol/L)	10.00 ± 6.33 ^a,^*	7.30 ± 3.04 ^b,^*	9.01 ± 10.55 ^b^	0.030	7.60 ± 3.21 ^a^	7.28 ± 2.82 ^a^	6.38 ± 1.63 ^a^	0.819
Sodium (mmol/L)	135.10 ± 7.40 ^a,^*	141.3 ± 2.92 ^a^	140.02 ± 3.44 ^a^	0.051	139.86 ± 9.21 ^a^	140.43 ± 2.23 ^a^	145.05 ± 4.34 ^a^	0.869
Potassium (mmol/L)	4.07 ± 0.64 ^a,^*	4.11 ± 0.35 ^a,^*	4.10 ± 0.15 ^a^	0.600	4.21 ± 0.58 ^a^	4.31 ± 0.40 ^a^	4.19 ± 0.39 ^a^	0.725
Chloride (mmol/L)	95.45 ± 7.57 ^a,^*	104.45 ± 3.44 ^b^	99.33 ± 4.90 ^a^	0.031	104.14 ± 8.21 ^a^	105.57 ± 2.37 ^a^	102.90 ± 4.32 ^a^	0.625

^†^*p*-values of the three related samples by Friedman’s two-way analysis of variance by ranks. Values with different superscripts (^a^ and ^b^) mean significant vs. pretransplant phase; * Significant versus the related samples in the women group by Mann–Whitney U test; BMI is body mass index; DEXA is dual-energy X-ray absorptiometry; BMD is bone mineral density; 25OHVD is 25 hydroxyvitamin D; ALP is alkaline phosphatase enzyme; Mg is magnesium.

**Table 3:** Bone mineral density and related biochemical parameters before, 1 year, and 2 years after the heart transplant between study groups.

Variables	Group I; 25OHVD < 25 nmol/L (*n* = 54)			*p*-Value ^†^	Group II; 25OHVD ≥ 25 nmol/L (*n* = 21)			*p*-Value ^†^
	Pre-Transplant Mean ± SD	1-y Post-Transplant Mean ± SD	2-y Post-Transplant Mean ± SD		Pre-Transplant Mean ± SD	1-y Post-Transplant Mean ± SD	2-y Post-Transplant Mean ± SD	
Weight (kg)	64.55 ± 9.22 ^a^	68.67 ± 12.04 ^b,^*	70.89 ± 9.67 ^b,^*	0.035	74.57 ± 26.57 ^a^	83.39 ± 17.79 ^b^	86.99 ± 19.35 ^b^	0.057
BMI (kg/m^2^)	23.42 ± 3.63 ^a^	24.92 ± 4.58 ^b,^*	26.21 ± 5.02 ^b,^*	0.037	27.60 ± 7.51 ^a^	31.17 ± 4.56 ^a^	32.67 ± 3.47 ^a^	0.063
DEXA parameters								
BMD lumbar spine (gm/cm^2^)	1.04 ± 0.14 ^a^	1.01 ± 0.16 ^a^	0.98 ± 0.26 ^a^	0.179	1.09 ± 0.15 ^a^	0.83 ± 0.11 ^a^	1.01 ± 0.16 ^a^	0.207
Lumbar spine Z-score	−0.19 ± 1.43 ^a^	−0.69 ± 1.28 ^b^	−0.45 ± 1.20 ^a^	0.002	−0.40 ± 1.37 ^a^	−0.84 ± 1.33 ^a^	−0.86 ± 1.86 ^a^	0.368
BMD femoral neck (gm/cm^2^)	0.86 ± 0.33 ^a^	0.71 ± 0.34 ^b^	0.85 ± 0.25 ^b^	0.001	0.96 ± 0.17 ^a^	0.79 ± 0.38 ^a^	0.97 ± 0.13 ^a^	0.867
Femoral neck Z-score	−0.06 ± 1.09 ^a^	−0.73 ± 0.96 ^b^	−0.49 ± 1.00 ^a^	0.001	−0.17 ± 1.58 ^a^	−0.27 ± 1.52 ^a^	−0.34 ± 1.35 ^a^	0.867
Biochemical parameters								
25OHVD (nmol/L)	11.52 ± 6.49 ^a,^*	48.49 ± 27.11 ^b^	64.00 ± 22.23 ^b^	<0.001	52.86 ± 16.41 ^a^	58.86 ± 38.52 ^a^	77.14 ± 38.33 ^a^	0.368
Vitamin D_3_ (nmol/L)	10.97 ± 5.84 ^a,^*	41.43 ± 24.84 ^b^	57.67 ± 16.67 ^b^	<0.001	33.29 ± 19.35 ^a^	32.19 ± 22.96 ^a^	76.14 ± 38.06 ^b^	0.032
Intact parathyroid h (pmol/L)	63.50 ± 51.29 ^a^	23.89 ± 39.54 ^a^	91.64 ± 43.51 ^b^	0.002	83.86 ± 58.24 ^a^	37.86 ± 35.92 ^a^	100.48 ± 45.78 ^a^	0.368
ALP (U/L)	91.28 ± 35.60 ^a^	79.39 ± 26.70 ^a^	71.01 ± 13.56 ^a^	0.056	125.14 ± 48.61 ^a^	112.43 ± 59.71 ^a^	96.43 ± 42.96 ^a^	0.066
Calcium (mmol/L)	2.16 ± 0.11 ^a,^*	2.26 ± 0.99 ^b^	2.25 ± 0.93 ^b^	0.016	2.27 ± 0.17 ^a^	2.18 ± 0.12 ^a^	2.15 ± 0.45 ^a^	0.565
Phosphate (mmol/L)	1.14 ± 0.33 ^a^	1.11 ± 0.22 ^a^	1.05 ± 0.17 ^a^	0.454	1.10 ± 0.27 ^a^	1.14 ± 0.25 ^a^	1.11 ± 0.30 ^a^	0.867
Mg (mmol/L)	0.88 ± 0.10 ^a^	0.72 ± 0.12 ^b^	0.62 ± 0.17 ^b^	<0.001	0.90 ± 0.14 ^a^	0.70 ± 0.10 ^a^	0.68 ± 0.11 ^a^	0.066
Creatinine (umol/L)	85.94 ± 28.05 ^a^	105.83 ± 55.69 ^a^	89.47 ± 25.01 ^a^	0.486	109.29 ± 41.92 ^a^	94.57 ± 32.35 ^a^	99.86 ± 22.70 ^a^	0.156
Urea (mmol/L)	8.06 ± 4.25 ^a^	7.31 ± 3.28 ^b^	5.93 ± 1.86 ^b^	0.076	13.29 ± 7.97 ^a^	7.26 ± 2.04 ^a^	15.06 ± 16.70 ^a^	0.102
Sodium (mmol/L)	134.50 ± 6.79 ^a^	141.44 ± 2.79 ^a^	143.12 ± 4.14 ^a^	0.251	139.71 ± 7.50 ^a^	140.43 ± 2.70 ^a^	143.15 ± 3.36 ^a^	0.867
Potassium (mmol/L)	4.04 ± 0.54 ^a^	4.18 ± 0.33 ^a^	4.07 ± 0.19 ^a^	0.600	4.29 ± 0.75 ^a^	4.01 ± 0.42 ^a^	4.09 ± 0.24 ^a^	0.867
Chloride (mmol/L)	95.72 ± 7.64 ^a^	104.83 ± 3.49 ^b^	98.29 ± 5.91 ^a^	0.131	98.29 ± 5.47 ^a^	104.14 ± 2.85 ^a^	102.97 ± 3.34 ^a^	0.867

^†^*p*-values of the three related samples by Friedman’s two-way analysis of variance by ranks. Values with different superscripts (^a^ and ^b^) mean significant vs. pretransplant phase; * Significant versus the related samples in Group II by Mann–Whitney U test; BMI is body mass index; DEXA is dual-energy X-ray absorptiometry; BMD is bone mineral density; 25OHVD is 25 hydroxyvitamin D; ALP is alkaline phosphatase enzyme; Mg is magnesium.

**Table 4:** Multivariable Cox proportional hazards regression model based on pretransplant variables.

Variables	Group I (25OHVD < 25 nmol/L)				Group II (25OHVD ≥ 25 nmol/L)			
	HR	95% CI		*p*-Value	HR	95% CI		*p*-Value
		Lower	Upper			Lower	Upper	
Age	0.962	0.888	1.042	0.345	0.869	0.696	1.086	0.218
BMI	1.327	1.015	1.733	0.038	1.145	1.756	1.734	0.524
Normal BMD at LS	Reference				Reference			
Osteopenia at LS	0.342	0.000	77,594.19	0.865	0.008	0.000	-	0.995
Osteoporosis at LS	0.000	0.000	-	0.995	0.000	0.000	-	0.995
Normal BMD at FN	Reference				Reference			
Osteopenia at FN	0.538	0.000	123,625.63	0.922	0.004	0.000	-	0.994
Osteoporosis at FN	0.000	0.000	-	0.996	0.382	0.000	-	0.999
VD_3_ serum level	0.916	0.712	1.179	0.497	0.958	0.868	1.058	0.394
25OHVD serum level	0.930	0.681	1.270	0.648	0.345	0.650	1.163	0.345

HR is hazard ratio; BMI is body mass index; BMD is bone mineral density; LS is lumbar spine; FN id femoral neck; 25OHVD is 25 hydroxyvitamin D; VD_3_ is vitamin D_3_.

## Data Availability

The raw data supporting the conclusions of this article will be made available by the authors, without undue reservation, to any qualified researcher.
